# Influence of low calcium availability on cadmium uptake and translocation in a fast-growing shrub and a metal-accumulating herb

**DOI:** 10.1093/aobpla/plv143

**Published:** 2015-12-07

**Authors:** Franziska Eller, Hans Brix

**Affiliations:** 1 Department of Bioscience, Aarhus University, Aarhus 8000, Denmark; 2 Present address: Biocenter Klein Flottbek, Hamburg University, Hamburg 22609, Germany

**Keywords:** *Brassica juncea*, cadmium toxicity, calcium, heavy metal, ion antagonists, phytoextraction, phytoremediation, *Sesbania sesban*

## Abstract

Calcium (Ca) and the toxic heavy metal cadmium (Cd) are antagonistic ions competing for uptake in plants when they co-occur in soil solutions, and high Ca concentrations can reduce the uptake of Cd in plants. However, less is known about the effects of low Ca bioavailability on Cd uptake and translocation in plants. We hypothesized that low Ca availability would enhance Cd uptake and translocation in *Sesbania sesban*, a fast-growing shrub potentially useful for Cd removal from contaminated soils, and *Brassica juncea*, a well-known Cd-hyperaccumulator. The two species were grown under controlled conditions for 21 days in hydroponic nutrient solutions with either 0.2 or 2 mM Ca and 0 or 50 µM Cd in a 2 × 2 factorial experimental design. Both species had a lower relative growth rate, final root length and shoot and root biomasses at 50 µM Cd compared with growth without Cd. The negative effects of Cd on both species were exacerbated at low Ca availability. *Brassica juncea* had higher root Cd concentrations than *S. sesban*, but the shoot Cd concentrations did not differ between the two species. The low Ca concentration enhanced the uptake of Cd in the roots of both species, but Cd translocation to the shoots was low, especially at 0.2 mM Ca. We conclude that the low Ca concentration enhanced the uptake of Cd into roots of *S. sesban* and *B. juncea* and increased the phytotoxicity of Cd. The translocation of Cd to the shoots of the two species was, however, lower at 0.2 mM than at 2 mM Ca, implying that Cd removal from polluted soil cannot simply be increased by adjusting ion concentrations.

## Abstract (precis)

We analyzed whether *low* calcium availability, as compared to high calcium availability, increased the accumulation of cadmium in *Brassica juncea* and *Sesbania sesban*. The cadmium uptake in the roots of both species was indeed increased, but not in the shoots. The translocation of Cd to the shoots is a complex process that seemed to be less affected by external ion concentrations and activities. As both species' viability was low under low calcium and high cadmium, the harvestable biomass limited the amount of Cd that could potentially be removed from polluted media by harvesting.

## Introduction

Cadmium (Cd) is an environmentally and sanitarily harmful heavy metal contaminant, resulting from anthropogenic sources such as industrial effluents, sewage sludge, phosphorus fertilizers and mining (Williams and David 1973; Molina *et al*. 2009; Schipper *et al*. 2011). Cadmium is water soluble, highly mobile and toxic even at relatively low concentrations (Das *et al*. 1997). As several metals are essential nutrient elements, plants have a natural disposition to take up minerals, including heavy metal. Transporters or channels in root membranes for essential ions have been determined as possible entry pathways for metals with unknown biological function, such as Cd, into plants (Clemens 2006). High Cd levels in plants disrupt ion homeostasis by interfering with the uptake of essential ions such as iron and magnesium, resulting in photosynthetic reduction, inhibition of the nitrogen metabolism and a decreased water and mineral uptake (Nascimento *et al*. 1998; di Toppi and Gabbrielli 1999).

Mineral elements with physically and chemically similar properties act as antagonists, and can replace each other by competition for the same uptake, transport and storage site within an organism (Hewitt 1949). For example, accumulation of Cd in the bark of *Populus canescens* has led to decreased calcium (Ca) concentration in the bark (He *et al*. 2013). In pea, iron deficiency has increased Cd transport and accumulation in roots and shoots (Cohen *et al*. 1998). Conversely, Cd uptake and translocation from roots to shoots were supressed in several plant species by adding other divalent cations, such as manganese, zinc or silicon, and especially Ca, to the growth solution (Jarvis *et al*. 1976; Sterckeman *et al*. 2011; Naeem *et al*. 2015). An increase in the Ca concentration in the soil solution has been shown to result in higher Ca ion activity at the root cell plasma membranes and, hence, in decreased uptake and toxicity of other cations (Kopittke *et al*. 2014). A non-selective uptake system for Ca^2+^ probably also mediates the entry of other cations, such as Na^+^ and Cd^2+^, across the plasmalemma (Clemens *et al*. 1998).

The Cd concentration of shoots of several plant species is positively correlated with Cd influx in roots (Stritsis and Claassen 2013). Moreover, the Cd concentration of the solution has been shown to be directly proportional to the shoot Cd concentration of several crop species, possibly due to passive influx of Cd through root membrane channels permeable to other divalent cations (Perfus-Barbeoch *et al*. 2002; Stritsis *et al*. 2012; Stritsis and Claassen 2013). Calcium plays a key role in plant development and cellular operations, due to its regulatory function for a range of proteins, gene expression and membrane transport systems (Bush 1995). Calcium is also involved in the synthesis of glutathione, a precursor of phytochelatin, thereby contributing to the immediate inactivation of metal ions entering the cytosol (Clemens 2006; López-Climent *et al*. 2014). An increase in Ca activity in growth solutions can also mitigate the deleterious effects of toxic ions other than Cd (Rengel 1992). In contrast, a low concentration of Ca may increase Cd uptake, due to lower competition and lower ion activity of Ca at the root uptake sites. Cadmium uptake into the whole plant was shown to be controlled by roots through absorption followed by xylem loading and translocation, with limited control of the accumulation by the shoot (Sterckeman *et al*. 2015). Absorption of Cd into roots occurs through both the symplastic and the apoplastic pathways, depending on the species, and it follows Michaelis–Menten kinetics (Redjala *et al*. 2012). Moreover, under exposure to high Cd, an apoplastic transport system for Ca and Zn to the shoot may contribute considerably to the root-to-shoot Cd transport (White 2001; White *et al*. 2002).

Most studies about mineral element interactions have so far dealt with high amounts of antagonistic ions exceeding the nutritional needs of the plants, since high concentrations may lower the bioavailability and activity of the target metal for phytoextraction (Issa *et al*. 1995; Osterås and Greger 2006; Ferreira *et al*. 2013; López-Climent *et al*. 2014). With the present study, we aimed at elucidating whether Cd uptake and translocation in plant tissues would be enhanced by low Ca availability. We investigated Cd uptake and translocation in Indian mustard (*Brassica juncea* (L.) Czern., Brassicaceae), which is a well-known metal accumulator and has been widely used as a model system for studying the physiological and biochemical pathways and consequences of metal accumulation in plants (Salt *et al*. 1995; Saraswat and Rai 2009; Mohamed *et al*. 2012). In addition, we used *Sesbania sesban* (L.) Merr. (Fabaceae), a fast-growing nitrogen-fixing shrub, which is widely distributed and cultivated throughout subtropical and tropical Africa and Asia. *Sesbania sesban* is commonly utilized as green manure, fuel wood and for forage production (Oosting *et al*. 2011). It has a very high biomass production, and is a promising species for the remediation of several metal pollutants (Chan *et al*. 2003; Yang *et al*. 2003; Gudu *et al*. 2009).

The accumulation of Cd in plant tissues has been shown to be one to two orders of magnitude lower when the plants are grown in soil, compared with growth in hydroponic media with similar Cd concentration (Kayser *et al*. 2000; Schwartz *et al*. 2003). Also, the Cd concentration at the root surface may differ from that of the soil solution and vary over time (Barber 1995), and the Cd availability may be affected by the ion composition in soil solutions (Stritsis *et al*. 2012; Stritsis and Claassen 2013). Since ion uptake from soils results from the interaction of soil and plant processes (Stritsis *et al*. 2014), and we were interested only in the plant processes affecting Cd accumulation, we chose to cultivate the two species in a hydroponic solution with stable Cd and high and low Ca. We hypothesized that Cd absorption, and hence the phytoextraction potential of *S. sesban* and *B. juncea*, would be enhanced when the plants were grown at low Ca availability. We showed that both *S. sesban* and *B. juncea* accumulated high concentrations of Cd in their above-ground and below-ground tissues. The low Ca availability indeed enhanced Cd uptake in the roots of both species, but not in the shoots. However, because growth and biomass production of both plant species were severely inhibited by Cd, especially at the low Ca concentration, phytoextraction of Cd by the two plant species under the studied conditions would not be feasible.

## Methods

### Plant growth and experimental set-up

Seeds of *B. juncea* and *S. sesban* were germinated and grown in commercial peat for 16 days in an indoor growth cabinet (BIO 2000S, Weiss Umwelttechnik GmbH, Lindensruth, Germany) at a relative air humidity of 80 %, a temperature of 27/25 °C (day/night), a light : dark cycle of 12 h and at an irradiance of ∼400 µmol m^−2^ s^−1^ photosynthetically active radiation at the base of the plants, provided by metal halide bulbs. After gently cleaning the roots, each seedling was transferred to a 1.4-L darkened glass container with hydroponic medium. The containers were randomly assembled and each container was provided with constant aeration.

The hydroponic medium was prepared in deionized water from a commercial macronutrient NPK fertilizer with magnesium (Pioner NPK Macro 19-2-15 + Mg Green, Brøste, Lyngby, Denmark), in the following final concentrations (in mM): 4.25 NO_3_, 2.64 NH_4_, 0.37 P, 1.97 K, 0.62 Mg and 0.61 S, adjusted to pH 6.5. Additionally, micronutrients were supplied (in µM: 0.02 B, 2.2 Cu, 24 Fe, 9.1 Mn, 0.5 Mo and 2.8 Zn) from a commercial micronutrient stock solution (Pioner Micro Plus with Fe, Brøste). Calcium was added as CaSO_4_ (1 mM) and CaCl_2_ (1 mM), resulting in a total Ca concentration of 2 mM in the solution. This concentration level is within the normal range (1–3 mM Ca) of the Ca concentrations used in other studies investigating heavy metal uptake in *B. juncea* and *S. sesban* (Ishikawa *et al*. 2006; Gudu *et al*. 2009; Mohamed *et al*. 2012; Suthar *et al*. 2013).

After an acclimation period of 9 days (Day 9) in this nutrient solution, the medium was renewed in all containers. Half of the plants were kept in the high Ca nutrient medium (2 mM Ca; high Ca treatment), while the other half of the plants were provided with the low Ca medium (0.2 mM Ca; low Ca treatment). At the same time, Cd was added to the hydroponic solutions as CdCl_2_ in concentrations of either 0 μΜ (control; ‘−Cd’ treatment) or 50 μM (‘+Cd’ treatment), respectively. This comparably high Cd concentration was chosen due to its measurable effects on root length growth and biomass production, following the results of a 15-day pilot experiment and also following other studies using high Cd concentrations (Gratão *et al*. 2008; López-Climent *et al*. 2014). The ion activities of Ca and Cd in the nutrient solution at pH 6.5 were calculated using the software SGCS (Speciation Gouy Chapman Stern) (Kopittke *et al*. 2014). The ion activity of Cd at the root cell plasma membrane in the high Ca solution was 45 times lower than that of Ca. The ion activity of Cd in the low Ca solution, however, was only four times lower than that of Ca. This was due to a 67 times higher Ca activity for 2 mM Ca compared with 0.2 mM Ca in the solutions. The Cd activity was, as a matter of fact, six times higher in the high than in the low Ca solution.

The experiment was a 2 × 2 × 2 factorial design, with two plant species, two Ca and two Cd concentrations with five replicates within each treatment, resulting in 20 plants per species. At Days 17 and 23 (*S. sesban*) or 24 (*B. juncea*), the hydroponic growth solutions in the containers were renewed. The plants were harvested after 30 days of growth, 21 of which were with the different Ca and Cd treatments.

### Final biomass, RGR and root length

The fresh mass of the plants was determined by a standardized weighing procedure and the maximum root length of each plant was measured at the beginning of the experiment (Day 1), at each solution renewal (Days 9, 17 and 23/24) and at harvest (Day 30). However, the root length of *B. juncea* at Day 24 was only assessed in the ‘0.2 mM Ca +Cd’ treatment, because the roots in the other treatments were entangled so a non-invasive root length measurement was impossible. The fresh mass to dry mass ratio of each species was estimated by oven-drying at 105 °C for ∼30 h of fresh plants similar to those used in the experiment. The ratio was used to convert the initial fresh mass to dry mass. The relative growth rate (RGR) of the plants was calculated as the natural logarithm of the difference in the final (Day 30) and initial (Day 9) dry masses, divided by the duration of the experimental treatments in days. The plants at Day 30 were separated into roots and shoots, and the dry masses of the fractions were determined.

### Analysis of Ca and Cd in tissues

At harvest, the plants were thoroughly rinsed with deionized water and the roots were separated from the shoots. The plant fractions were thereafter oven-dried at 105 °C for ∼30 h. The Ca and Cd concentrations in the plant tissues were then analysed by inductively coupled plasma emission spectrometry (Optima 2000 DV, Perkin Elmer, USA) after digestion of the ground plant material in HNO_3_–HCl–H_2_O_2_ in a microwave oven (Multiwave 3000, Anton Paar GmbH, Austria). The translocation factor (TF) was calculated as the ratio between the Cd concentration in the shoot and the Cd concentration in the root of the plant.

### Data analyses

All data were analysed by Statgraphics Centurion XVI.I (Statpoint Technologies Inc., Warrenton, VA, USA). The data for the dry mass, RGR, final root length and tissue concentrations of Ca were analysed using the General Linear Models procedure with ‘Species’, ‘Ca treatment’ and ‘Cd treatment’ as main factors. Since the tissue Cd concentrations in all −Cd treatments were below the detection limit, these parameters as well as the TF were analysed by two-way analysis of variance (ANOVA) with ‘species’ and ‘Ca treatment’ as the main factors. To detect differences due to treatment effects, *post hoc* comparisons of means were made by the Tukey's honestly significant differences procedure. If necessary to improve normality and homoscedasticity, data were log- or sqrt-transformed. One *B. juncea* replicate of the ‘0.2 mM Ca −Cd’ treatment was accidentally damaged and died before the end of the experiment; hence, it was removed from all statistical analyses.

## Results

### Shoot and root biomass, RGR and root length

**Figure 1.**
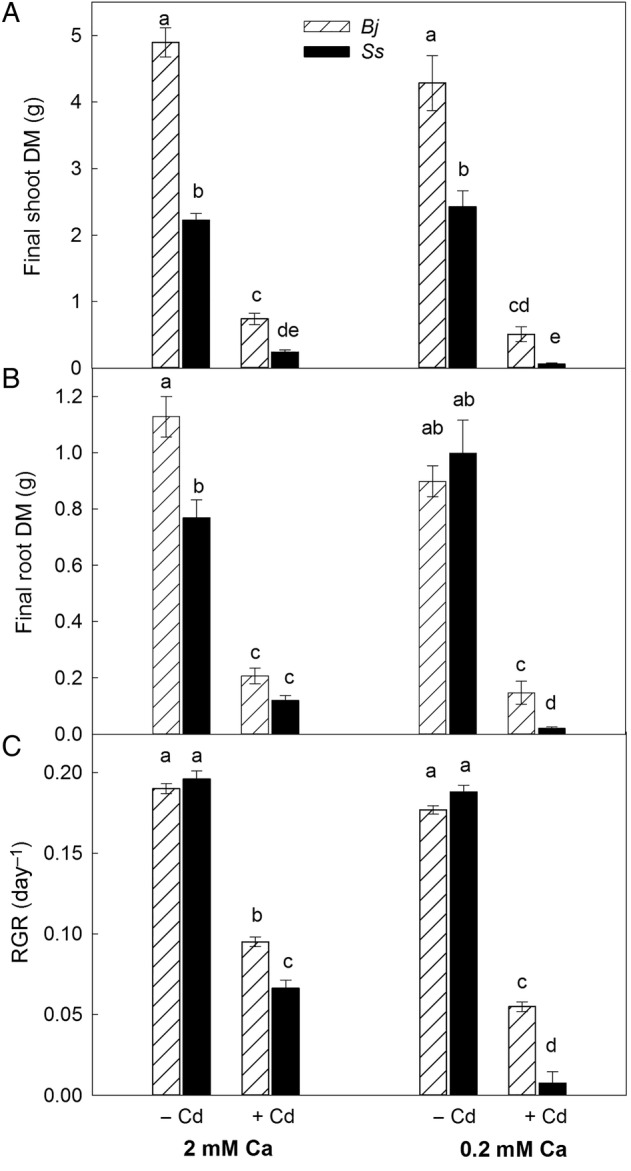
Mean (±1 SE) final dry mass of shoots (A), roots (B) and RGR (C) of *B. juncea* (*Bj*) and *S. sesban* (*Ss*) during 21 days of hydroponic growth in different treatment combinations of Ca (0.2 and 2 mM Ca) and Cd (0 µM, −Cd and 50 µM, +Cd). Different letters within the same panel indicate significant differences between the treatments.

*Brassica juncea* had two to three times higher shoot biomass production than *S. sesban* across all treatments, but the difference was especially obvious under Cd exposure, where *B. juncea* produced up to eight times more shoot biomass than *S. sesban* (significant species × Cd interaction, Table 1, Fig. 1A). In both species, Cd exposure resulted in a severe reduction of both shoot and root biomass. The high Ca availability resulted in somewhat higher shoot biomass production of both species under Cd exposure (weak Ca × Cd interaction; *P* = 0.06, Table 1). The two species responded distinctly with root biomass production to both Cd and Ca, as shown by the significant three-way interaction (Table 1). In general, the response to high Ca availability was higher biomass production when plants were exposed to Cd, and this response was specifically pronounced in roots of *S. sesban* (Fig. 1A and B). Under Cd exposure, *B. juncea* had significantly higher RGR than *S. sesban*, as shown by the significant species × Cd interaction. Exposure to Cd negatively affected the RGR of both species, and the effect was more profound when the plants were grown at the low Ca concentration (significant Ca × Cd interaction; Table 1, Fig. 1C).

**Table 1.** *F*-ratios from general linear model analyses and two-way ANOVAs showing the effects of species (*B. juncea* vs. *S. sesban*), Ca concentration of the growth medium (0.2 vs. 2 mM Ca) and Cd concentration of the growth medium (0 vs. 50 µM Cd) and their interactions on final shoot and root dry mass (DM), RGR, final root length, Ca and Cd concentrations in shoots and roots and the TF in the two plant species. df, degrees of freedom. Significance level (in bold): *P < 0.05, **P < 0.01, ***P < 0.001.

Parameter	Main factors			Interactions			
	Species (df = 1)	Ca (df = 1)	Cd (df = 1)	Spec × Ca (df = 1)	Spec × Cd (df = 1)	Ca × Cd (df = 1)	Spec × Ca × Cd (df = 1)
Final shoot DM	157.2***	8.94**	934.1***	0.5	5.8*	3.7	3.3
Final root DM	20.4***	7.1*	595.8***	1.1	3.5	7.6**	11.4**
RGR	21.0***	86.8***	1680***	1.2	51.7***	35.7***	3.5
Final root length	18.3***	8.0**	60.0***	2.4	4.7*	6.5*	2.3
Ca_shoot_	80.6***	219.0***	13.9**	3.7	0.5	0.7	0.0
Ca_root_	29.8***	131.3***	62.6***	70.7***	34.4***	11.2**	1.1
Cd_shoot_	1.7	1.5	–	2.5	–	–	–
Cd_root_	64.6***	86.2***	–	5.4	–	–	–
TF	6.7*	13.4**	–	5.9*	–	–	–

**Figure 2.**
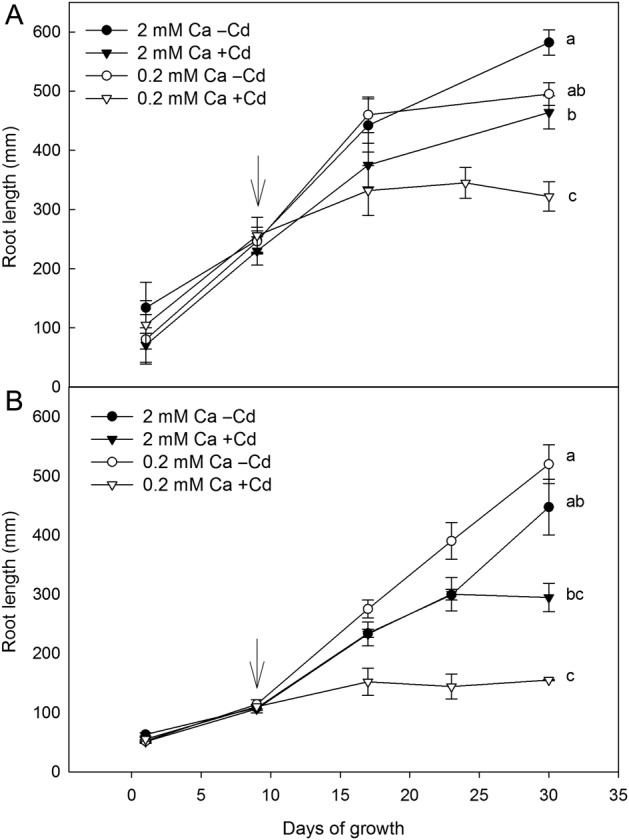
Root length (mean ± 1 SE) of *B. juncea* (A) and *S. sesban* (B), hydroponically grown in different treatment combinations of Ca (0.2 and 2 mM Ca) and Cd (0 µM, −Cd and 50 µM, +Cd). Arrows indicate the start of the Cd treatments. Different letters on the final root length within the same panel indicate significant differences between the treatments.

The roots of both species were consistently shorter when exposed to Cd within the same Ca treatment (Fig. 2). In the +Cd treatment, *B. juncea* had longer roots than *S. sesban* (significant species × Cd interaction; Table 1, Fig. 2). Also, in the +Cd treatment, the high Ca concentration reduced the Cd toxicity, as evidenced by longer roots of both species compared with the low Ca concentration (significant Ca × Cd interaction; Table 1). In the high Ca treatment, *S. sesban* had similar root lengths independent of Cd treatment until Day 23 (Fig. 2B). However, plants in the +Cd treatment were chlorotic and had lower growth rates compared with plants in the −Cd treatment.

### Tissue Ca and Cd concentrations

*Brassica juncea* had significantly higher Ca concentrations in shoots than *S. sesban* across all treatments. Also, the shoot Ca concentrations of both species were significantly higher at 2 mM Ca compared with 0.2 mM Ca, and were also slightly higher in the +Cd, compared with the −Cd treatments (Tables 1 and 2). *Brassica juncea* had higher root Ca concentrations than *S. sesban* in the high Ca treatments and in the 0.2 mM Ca −Cd treatment. *Sesbania sesban* had higher root Ca concentration when exposed to Cd within the same Ca treatment, but this was not true for *B. juncea* (significant species × Ca and species × Cd interaction; Tables 1 and 2). In general, growth at 2 mM Ca resulted in a higher Ca concentration of the roots of both species (Table 2).

**Table 2.** Calcium and Cd concentrations (mean ± 1 SE) in tissues of *B. juncea* (*Bj*) and *S. sesban* (*Ss*) after 21 days of growth in different treatment combinations of 0.2 and 2 mM Ca, and 0 µM (−Cd) and 50 µM (+Cd) Cd, respectively. Different letters within the same column indicate significant differences between the treatments. DM, dry mass.

Treatment	Species	Ca shoot (mg g^−1^ DM)	Ca root (mg g^−1^ DM)	Cd shoot (mg g^−1^ DM)	Cd root (mg g^−1^ DM)
2 mM Ca –Cd	*Bj*	33.6 ± 1.6^a^	7.2 ± 0.4^a^	–	–
	*Ss*	15.5 ± 0.6^b^	3.3 ± 0.3^c^	–	–
0.2 mM Ca −Cd	*Bj*	8.9 ± 0.1^c^	2.5 ± 0.1^cd^	–	–
	*Ss*	5.7 ± 0.4^d^	1.7 ± 0.1^d^	–	–
2 mM Ca +Cd	*Bj*	43.3 ± 1.1^a^	7.2 ± 0.2^a^	1.0 ± 0.0	3.7 ± 0.2^b^
	*Ss*	18.4 ± 0.2^b^	5.3 ± 0.2^b^	0.6 ± 0.0	2.1 ± 0.2^c^
0.2 mM Ca +Cd	*Bj*	14.4 ± 3.0^bc^	3.5 ± 0.2^c^	1.0 ± 0.2	7.0 ± 0.3^a^
	*Ss*	7.4 ± 0.0^cd^	5.5 ± 0.0^ab^	1.0 ± 0.0	4.1 ± 0.0^b^

There were no significant differences in shoot Cd concentrations between the species or Ca treatments (Tables 1 and 2). However, *B. juncea* had significantly higher Cd concentrations in roots than *S. sesban*, and roots of both species contained higher Cd concentrations in the low Ca than in the high Ca treatment (Tables 1 and 2).

### Translocation factors

The TF was calculated as the ratio between the Cd concentration in the shoot and the Cd concentration in the root of the plant. All TFs were <1. In the low Ca treatments, *S. sesban* had significantly higher TF (0.25 ± 0.01) than *B. juncea* (0.14 ± 0.02), whereas both species had similar TF in the high Ca treatment (*S. sesban*: 0.28 ± 0.01; *B. juncea*: 0.27 ± 0.01), as shown by the significant species × Ca interaction (Table 1). The high Ca concentration resulted in significantly higher TF of *B. juncea* only.

## Discussion

The plant bioavailability of metals such as Cd is highly affected by co-occurring ions in the soil solution. Calcium, a prevailing cation in many soils, has a diameter and valence similar to Cd, and may affect the Cd uptake from the soil into the plant. It has recently been shown that a high Ca concentration in the soil solution leads to a drastic alleviation and even neutralization of Cd stress in several Fabaceae species (Ferreira *et al*. 2013). This highlights the importance and efficiency of Ca against Cd toxicity. A disruption of the Ca homeostasis, e.g. as a consequence of competition with non-essential divalent ions, will lead to severe consequences such as impaired signalling and/or enzymatic processes. For our study, it was therefore important that the plants in the low Ca treatment were not suffering from Ca deficiency, which would have impaired their growth. The similar RGR, biomass production and final root length as well as the absence of chlorosis in the control plants of *S. sesban* and *B. juncea* in both Ca treatments proved that the plants were healthy and did not suffer from Ca deficiency.

We hypothesized that both species would accumulate a higher concentration of Cd when they were grown at relatively low Ca availability. The calculated ion activity of Cd at the root uptake site (Kopittke *et al*. 2014) was strongly diminished compared with the activity of Ca in the high Ca medium, but much less so in the low Ca medium. *Sesbania sesban* accumulated more Cd in its shoots at low than at high Ca, but this was statistically not significant. The low Ca availability only enhanced Cd uptake in the roots of both species. Hence, our hypothesis was only partly confirmed by the results of our study. Similar results have been found for a non-hyperaccumulating ecotype of *Sedum alfredii*, which had higher Cd influx in roots with decreasing Ca concentration in the medium (Lu *et al*. 2010). For hyperaccumulating plants, there seems to be a low Ca threshold concentration in the medium, above which decreasing Ca concentrations result in accelerated Cd uptake. Very low Ca concentrations below this threshold will, in turn, inhibit Cd accumulation (Lu *et al*. 2010; He *et al*. 2015).

The transport of Cd from roots to shoots is low in most plant species (Jarvis *et al*. 1976), and also in the two species studied here. After Cd absorption in the root symplasm, further transport to the xylem is thought to be restricted by the production of phytochelatins and the subsequent sequestration of the Cd-chelate complexes in root vacuoles (Lux *et al*. 2010). Apoplasmic movement of Cd to the xylem can be restricted by the development of extracellular barriers, such as the exodermis and endodermis (Lux *et al*. 2010). The retention or immobilization of Cd in the roots is crucial for the protection of the photosynthetic apparatus (Mohamed *et al*. 2012). Adding chelating agents such as ethylenediaminetetraacetic acid can assist the Cd transport from roots to shoots (Lux *et al*. 2010), but we did not apply chelating substances in the present study, which may be an explanation for the low translocation found in this study.

Another possible reason for the low Cd translocation to shoots may have been the enhanced Ca accumulation in shoots under Cd exposure, even when Ca availability was low. *Brassica juncea* accumulated more Ca than *S. sesban* in its shoots, and also had higher Ca concentration in roots when not exposed to Cd. The high shoot and root Ca concentration may partly explain the higher resistance of *B. juncea* to Cd toxicity compared with *S. sesban*, which was demonstrated by its higher RGR and longer final root lengths in the +Cd treatments. Exposure to Cd has previously been shown to result in the down-regulation of enzymes involved in the detoxification of reactive oxygen species, and this down-regulation was reverted by Ca supply (Rodriguez-Serrano *et al*. 2009). Higher Ca concentration may, therefore, affect the transcriptional regulation of antioxidant defence mechanisms.

Cd toxicity can impair the stomatal conductance, partly because Ca channels, which play a key role in controlling guard cell regulation, are permeable to Cd (Hinkle *et al*. 1992). However, high tissue Ca concentrations can counteract Cd-induced stomatal closure, which would otherwise lead to decreased CO_2_ fixation (McAinsh *et al*. 1997; Perfus-Barbeoch *et al*. 2002). In *S. sesban*, higher root Ca concentrations were associated with lower root Cd concentrations compared with *B. juncea* at 0.2 mM Ca. A similar inverse relationship between Ca concentration of the growth solution and root Cd concentrations has been found for maize (Sterckeman *et al*. 2011), *Picea abies* seedlings (Osterås and Greger 2006) and for the green alga *Kirchneriella lunaris* (Issa *et al*. 1995).

For efficient Cd phytoextraction from the soil, the metal must be concentrated in the harvestable parts of the plants, since these are the plant parts that can be disposed of. The TF has been used as an indicator of the ability of plants to translocate metals to the above-ground harvestable tissues. Plants possessing a TF > 1 are generally considered suitable for phytoextraction (Baker and Brooks 1989; Saraswat and Rai 2009). The low TFs (<1) for Cd of both *B. juncea* and *S. sesban* suggest that these species are less suitable for Cd remediation, at least at high Cd concentrations as in our experiment. The low TF for Cd found for *B. juncea* in this study is not in agreement with results from several other studies reporting TF for Cd greater than one (Salt *et al*. 1995; Jiang *et al*. 2004; Su and Wong 2004; Saraswat and Rai 2009; Mohamed *et al*. 2012; Suthar *et al*. 2013). However, in most of the cited studies, the Cd bioavailability and accumulation in the plant tissues, and thus, toxicity, were lower than in the present study.

Both *B. juncea* and *S. sesban* showed impaired growth and reduced biomass production under Cd toxicity, which are incompatible with the high biomass production required for successful phytoextraction. We can, however, not rule out—especially under the low supply of Ca—that the uptake of other essential nutrients may have been restricted because of the competition with Cd. This may have further exacerbated the detrimental effects of Cd, as previously shown for *B. juncea* (Ahmad *et al*. 2015).

## Conclusions

We have shown that a low availability of 0.2 mM Ca enhanced the uptake of Cd in roots of both *S. sesban* and *B. juncea*, compared with the Cd uptake at a 10 times higher Ca concentration. However, Cd translocation to the above-ground tissues of both species was generally low and even lower at 0.2 mM than at 2 mM Ca in the growth medium, especially in *B. juncea*. Furthermore, the growth and biomass production of both plant species were reduced at low Ca bioavailability, indicating that these species would not be suitable for phytoextraction of Cd under the studied conditions of low Ca and high Cd bioavailability. Hence, although both *B. juncea* and *S. sesban* accumulated high tissue Cd concentrations, the translocation of Cd to the harvestable biomass limited the amount of Cd that could potentially be removed by harvesting. The interaction of Cd with Ca as competing ion was important only for Cd uptake and accumulation in the roots, the site where the competition occurred. The translocation of Cd to the shoots is a complex process that seemed to be less affected by external ion concentrations and activities. Our study showed, however, that there may be possible effects of the Ca concentration in the plant tissue on Cd toxicity and transport to shoots, which are worth further investigation.

## Sources of Funding

This research was funded by a grant to H.B. from The Danish Council for Independent Research | Natural Sciences.

## Contributions by the Authors

F.E. and H.B. designed the experiment, F.E. conducted the experiment and both authors wrote the manuscript.

## Conflict of Interest Statement

None declared.
